# Work and Functional Limitations for Older Workers: Does Measurement Make a Difference?

**DOI:** 10.1093/geroni/igaa057.1492

**Published:** 2020-12-16

**Authors:** Theresa Andrasfay, Anne Pebley, Noreen Goldman

**Affiliations:** 1 Princeton University, Princeton, New Jersey, United States; 2 University of California Los Angeles, Los Angeles, California, United States

## Abstract

Social scientists have become increasingly interested in strenuous jobs as contributors to health inequality over the life course. Physically demanding work at later ages is of particular interest because it can have implications for retirement decisions, physical functioning, and disability, and strenuous jobs are prevalent among lower-income and minority older workers. Many studies have relied on occupational characteristics from the Occupational Information Network (O*NET), but few have assessed how these measures compare to self-reported occupational characteristics in terms of identifying social gradients in exposure and predicting future health outcomes. Using data from 16,683 respondents of the Health and Retirement Study, we obtained self-reported and O*NET measurements of general physical activity, frequency of lifting objects, and frequency of stooping/crouching required in the jobs they held at first interview. Pearson correlation coefficients revealed moderate correlations between the self-reported items and corresponding O*NET items. Though they are measured on different scales, both the self-reported and O*NET measures of physical demands revealed similar racial/ethnic and gender gradients in exposure to physically strenuous work. Lastly, we fit a series of random effects Poisson models to assess how these measures predict accumulation of functional limitations, a health outcome thought to result in part from strenuous working conditions. We found that while models using self-reported working conditions have the best fit with the data, models using the corresponding items in O*NET have comparable goodness-of-fit. These results suggest that, in the absence of self-reported physical occupational characteristics, O*NET characteristics provide a reasonable alternative.

